# Supplementary material for: HIV-1 Accessory Proteins Impart a Modest Interferon Response and Upregulate Cell Cycle-Related Genes in Macrophages
Source: Pathogens. 2022 Jan 26;11(2):163. doi: 10.3390/pathogens11020163 (PMC8878269; doi:10.3390/pathogens11020163)
Supplement: Supplementary file 1 [file pathogens-11-00163-s001.zip › pathogens-1488757-supplementary.pdf]

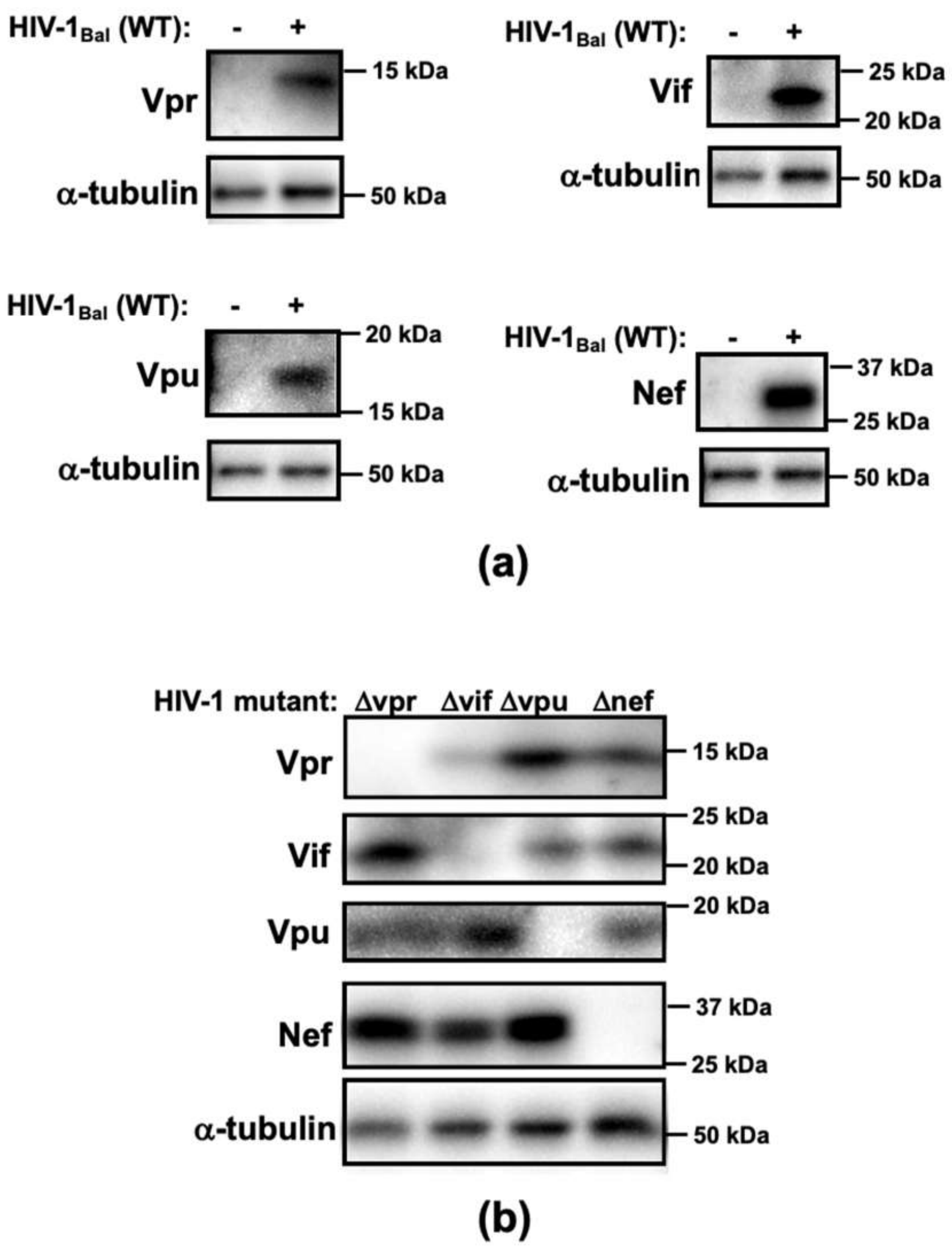

Figure S1. Western blot analysis of protein lysates from MDMs infected with wild-type (WT) and HIV-1$_{Bal}$ mutants from cell culture aliquots cultured in parallel with cultures harvested 24 hours post infection and subjected to RNA-seq (**a**) MDMs mock infected (left lane each blot) or infected with WT HIV-1$_{Bal}$ (right lane each blot). (**b**) western blots from protein extracts from HIV-1 deletion mutant infected cells harvested 24 hours post infection and in parallel to cultures subjected to RNA-seq.

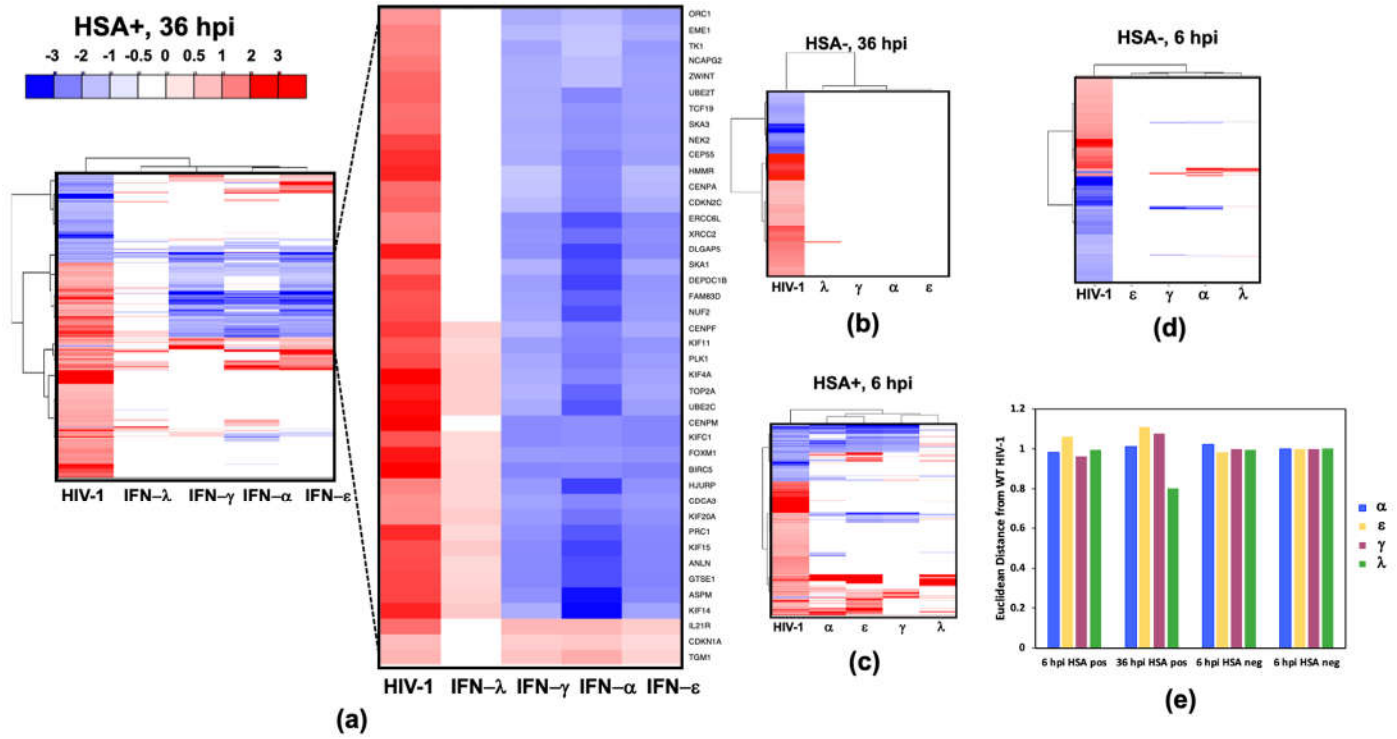

Figure S2. Comparison of transcriptome changes from resulting from HIV-1 infection from dataset published by Deshiere et al. in 2017 [33] to Type -I, -II, and -III IFN treatment. Heat map clustering of RNA-seq DEGs from the following comparisons (from left to right, WT HIV-1$_{Bal}$ (WT) vs mock infection, IFN-λ vs untreated, IFN-γ vs untreated, IFN-α vs untreated, IFN-ε vs untreated (P_{adj}<0.05, log2FC <1 or >1) HIV-1 vs mock conditions are as follows: (**a**) mCD24+ MDMs purified 36 hpi (**b**) mCD24- MDMs purified 36 hpi (**c**) mCD24+ MDMs purified 6 hpi (**d**) mCD24- MDMs purified 6 hpi

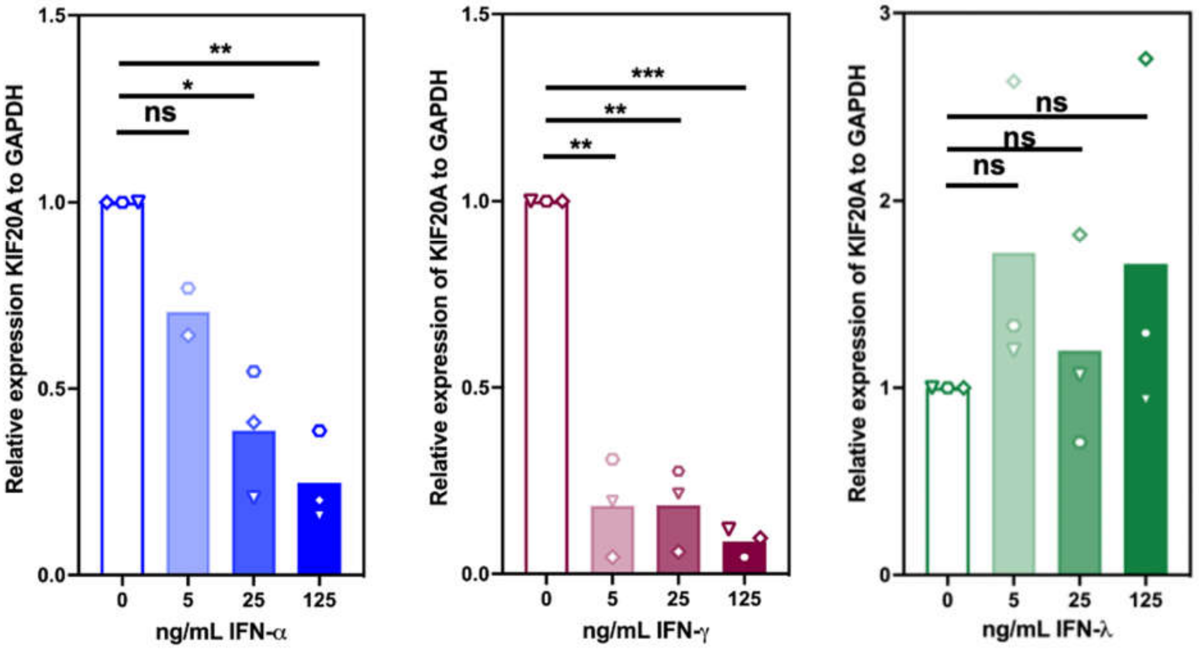

(a) **(b)** **(c)**

Figure S3. KIF20A expression in MDMs stimulated with IFN-α, IFN-γ, or IFN-λ. MDMs from three blood donors were stimulated with 0, 5, 25 or 125 ng/mL of IFNs for 18 hours. RNA was harvested and subjected to RT-PCR for measurement of KIF20A and GAPDH1 expression. Gene expression was determined using the ΔΔCt method. KIF20A expression levels are normalized to untreated samples and then to GAPDH expression. p-values computed using a 2 tailed paired student t test and significance assigned as follows: * : p-value < 0.05, **: p-value < 0.01, ***: p-value < 0.001, ns: p-value > 0.05.
